# PPAR-Alpha Agonist Used at the Acute Phase of Experimental Ischemic Stroke Reduces Occurrence of Thrombolysis-Induced Hemorrhage in Rats

**DOI:** 10.1155/2015/246329

**Published:** 2015-05-27

**Authors:** Sophie Gautier, Thavarak Ouk, Maud Pétrault, Olivier Pétrault, Vincent Bérézowski, Régis Bordet

**Affiliations:** ^1^U1171, Departement de Pharmacologie Medicale, University Lille Nord de France, Faculté de Médecine, CHU Lille, 1 Place de Verdun, 59037 Lille Cedex, France; ^2^Faculté de Médecine, Université de Lille 2, Lille, France; ^3^Centre Hospitalier Universitaire, Lille, France; ^4^Institut de Médecine Prédictive et de Recherche Thérapeutique, Lille, France; ^5^University d'Artois, 62307 Lens, France

## Abstract

The impact of fenofibrate, a peroxisome proliferator-activated receptor-alpha (PPAR-*α*) agonist, on the risk of thrombolysis-induced hemorrhage during the acute phase of stroke in a rat model of stroke was studied. One-hour middle cerebral artery occlusion followed by thrombolysis with tissue plasminogen activator was made in rats receiving either fenofibrate or vehicle for 72 h after stroke. Evaluation of infarct, hemorrhage, middle cerebral artery vasoreactivity, and immunochemistry (CD11b for microglial activation, myeloperoxidase, and ICAM-1 for neutrophil infiltration) was performed. The PPAR-alpha agonist significantly reduced the risk of hemorrhage after thrombolysis in parallel with a decrease in the infarct volume and in the stroke-induced vascular endothelial dysfunction. These effects are concomitant with a reduction in microglial activation and neutrophil infiltration in infarct area. Our results strengthen the idea that using drugs such as fenofibrate, with pleiotropic properties due to PPAR-alpha agonism, may be of value to reduce thrombolysis-induced hemorrhage during acute stroke.

## 1. Introduction

Thrombolysis with tissue plasminogen activator (tPA) is currently the only available medical treatment for acute ischemic stroke, allowing reperfusion of the infarcted area and improving the long-term functional outcome. However, tPA-therapeutic window is limited to 4,5 hours after stroke onset, due to an increase risk of intracerebral hemorrhages (ICH), a severe and unpredictable complication. As a consequence, less than 10% of patients are treated with this drug [1] and this situation justifies further research into new effective or adjunctive pharmacological strategies for stroke with thrombolysis [2] in accordance with the Stroke Therapy Academic Industrial Roundtable (STAIR) guidelines.

Activation of the peroxisome proliferator-activated receptors (PPARs) results in pleiotropic effects that target the whole neurovascular unit [3]. The PPAR-*α* isoform is of particular interest because of its pharmacological action on oxidative stress, inflammation, and leukocyte-endothelium interactions, all involved in ischemic stroke. We recently demonstrated that experimental PPAR-*α* modulation by fenofibrate, a direct synthetic ligand of PPAR-*α* isoform, generates beneficial effects on the immediate poststroke consequences [4]. The objective of this work was to test if the PPAR-alpha agonist fenofibrate administered as an adjunctive treatment during the acute phase of an experimental ischemic stroke in rats can prevent thrombolysis-induced hemorrhage.

## 2. Methods

All animal experiments were performed in strict accordance with the guidelines published by the international European ethical standards (86/609-EEC), the French Department of Agriculture (decree 87/848), and the local ethics committee of Nord-Pas de Calais. Spontaneously hypertensive rats (SHRs; 10-week-old male, weighing 270 to 320 g, from Elevage Janvier, France) were used.

Animals were housed in a light- and temperature-controlled environment with unlimited access to food and water.

### 2.1. Drug

Fenofibrate (F-6020; Sigma-Aldrich Chimie, Lyon, France) was dissolved in vehicle (water; 0.1% tween 80; 0.5% carboxymethylcellulose) and orally administered after stroke by gavage twice a day for 72 h.

### 2.2. Surgical Procedure and Design of the Study

Cerebral infarction was induced by intraluminal middle cerebral artery occlusion (MCAO) during 60 min as previously described [5]. Briefly, after anesthesia (chloral hydrate; 300 mg/kg, 1.7 mL), a monofilament nylon suture was inserted into the right external carotid artery to the internal carotid artery and then passed into the intracranial circulatory system as far as in the narrow lumen at the start of the MCA, leading to an occlusion of this artery. After 1 hour, the suture was carefully removed to allow reperfusion. To reproduce the conditions of thrombolysis and induced hemorrhages, tPA 10 mg/kg (6 mL/kg) was administered after its* in vitro* application for 30 minutes on a clot (made from 0.2 mL of autologous blood sampled by jugular vein during the surgery and left in the open air for 5 hours, allowing thrombus formation). The resulting solution (mainly contained plasmin) was collected and infused 5 hours after restoring cerebral blood flow, according to previously validated method [6]. All rats were submitted to MCAO and tPA treatment.

The study included all animals that underwent the whole protocol (72 h) and excluded nonischemic animals or animals with subcortical infarcts (10%). Two groups of animals were randomly formed and received* per os* fenofibrate (F-6020; Sigma-Aldrich Chimie, Lyon, France; 50 mg/kg/day) or vehicle (water; 0.1% tween 80; 0.5% carboxymethylcellulose), administered after stroke through a gavage performed twice a day during 72 h, with the first dose given 1 h after ischemia onset (*n* = 13 and *n* = 12, resp.). Experimental data were monitored by blinded investigator for group allocation.

### 2.3. Evaluation of Infarct and Hemorrhage

Seventy-two hours after restoring blood flow, animals were sacrificed with an overdose of intraperitoneal pentobarbital (200 mg/kg). The brains were rapidly removed and frozen. Coronal, 50 *μ*m thick slices were taken from 12 levels, according to Paxinos and Watson's stereotaxic atlas. Infarct volume (in mm^3^ and corrected for edema) was quantified by digital integration of the respective ischemic areas on all sections in a given animal after staining by cresyl fast violet (Color Image 1.32, NIMH, Bethesda, MD, USA). Intracerebral hemorrhages were assessed by blind histological evaluation on three defined sections (+0.48, −0.92, and −3.30 mm relative to the bregma). The incidence of ICH was scaled according to a previously described method (Gautier et al., 2009): 0 = no hemorrhage; 1 = multiple, macroscopically visible hemorrhages, seen as petechiae; and 2 = hematoma. The severity of the ICH was deemed to correspond to the number of petechial hemorrhages or hematoma per infarct area.

### 2.4. Vasoreactivity Analysis

Endothelium-dependent relaxation was assessed after 72 h of blood flow restoration [4] in a Halpern arteriograph (Living Systems Instrumentation, Burlington, VT, USA). A proximal segment of the right MCA was perfused with oxygenated Krebs solution and maintained at 37°C and pH 7.4. The experiment was performed under no-flow conditions. The lumen diameter was measured using image analysis. The relaxant dose-response curve for acetylcholine (Ach) was determined by stepwise, cumulative addition (from 0.001 to 10 *μ*M Ach). Relaxant responses were expressed as the percent increase in the preconstricted artery diameter.

### 2.5. Immunohistochemistry

After 72 h of reperfusion, the rats were anesthetized and perfused through the heart with cold saline and 4% paraformaldehyde. After 24 h of fixation in 4% paraformaldehyde at 4°C, the brains were cryoprotected in 20% and 30% sucrose solutions in phosphate-buffered saline (PBS) at 4°C. After fixation, brain sections (+0.48 mm relative to the bregma; 20 *μ*m thick) were blocked with 10% normal goat serum and incubated overnight at 4°C with mouse anti-rat CD11b (1 : 500; MCA275R, Serotec), rabbit polyclonal anti-MPO (myeloperoxidase; 1 : 500; A0398, DAKO), and mouse anti-rat ICAM-1 (1 : 500; MCA773, Serotec) versus brain sections incubated without antibodies (negative control). Next, sections were incubated with biotinylated horse anti-mouse IgG for CD11b and ICAM-1 and anti-rabbit IgG for myeloperoxidase for 3 h at room temperature, followed by treatment with an avidin-biotinylated enzyme complex (ABC kit, Vector Laboratories) and lastly diaminobenzidine tetrahydrochloride. Neutrophil infiltration and microglial activation were evaluated by counting positive cells on six adjacent, 1 mm^2^ fields in the ischemic zone (representative of 70 to 90% of the sliced ischemic tissue, located in cortical, subcortical, and striatal structures). As controls, we used brain sections of sham rats, submitted to surgery without MCAO and treated with vehicle.

### 2.6. Statistical Analysis

Based on the common standard deviation and on pilot experimental data using this model, we calculate the sample size needed to reject the null hypothesis to achieve an 80% power (two-tailed alpha = 0.05) to detect a 50% difference in hemorrhage and 30% in stroke infarct size. The number of rats per group at these criteria was determined to be at least seven. All values were expressed as mean ± standard error mean (SEM).

Statistical analysis was performed using SPSS 12.0 software. Comparisons among multiple groups were performed using one-way analysis of variance followed by a post hoc Protected Least Significant Difference Fisher test if variance analysis was significant post hoc. Comparisons between two groups were achieved using Mann-Whitney test. A value of *p* < 0.05 was considered as statistically significant.

## 3. Results

For physiologic parameters (weight, blood pressure, temperature, pH, PaCO_2_, and PaO_2_), no difference was seen between vehicle and fenofibrate-treated groups before, during, and after the surgical procedure.

### 3.1. Effect of Fenofibrate on tPA-Induced Hemorrhages

**Figure 1:**
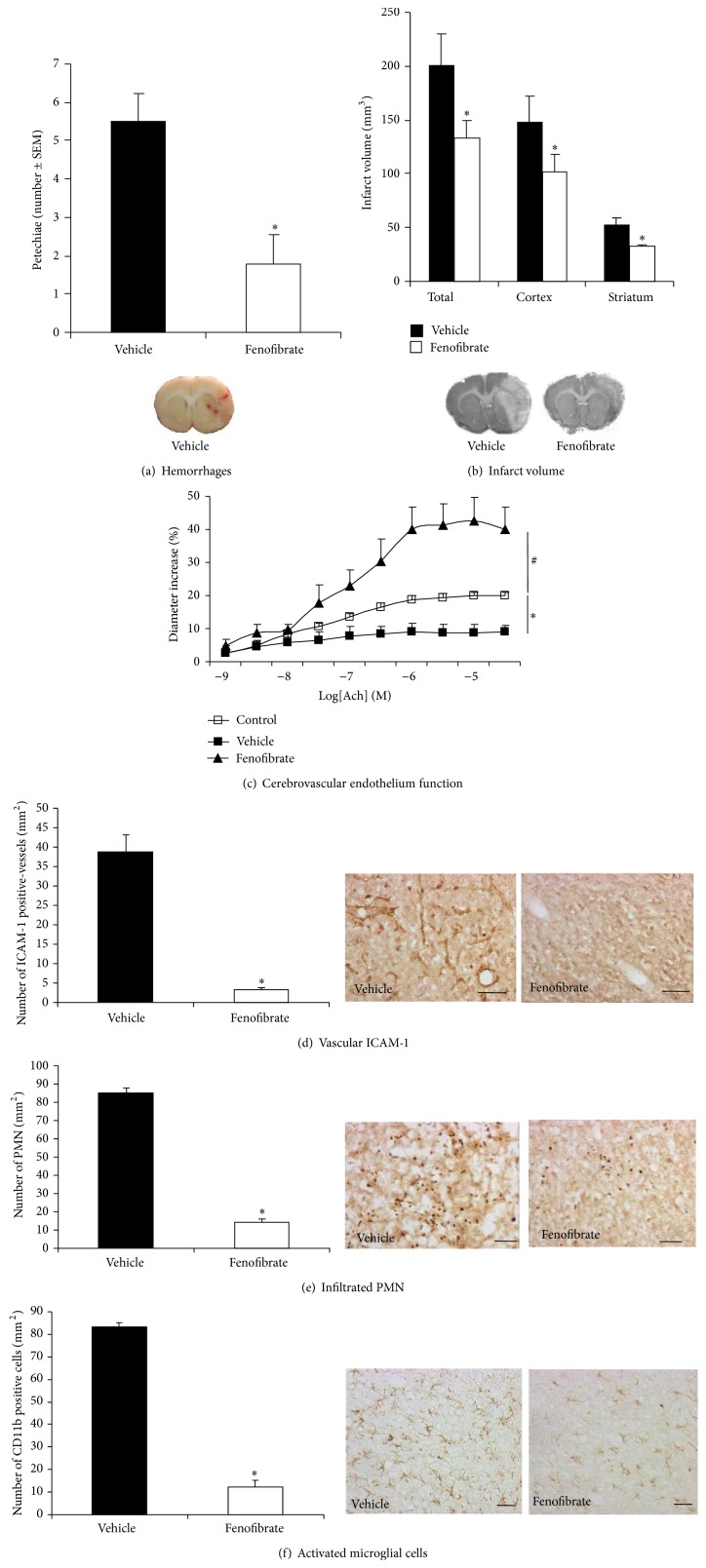
Evaluation of the effects of the PPAR-*α* agonist fenofibrate administered during the acute phase of experimental stoke in combination with thrombolysis with tPA on neuronal and vascular compartments. Evaluation takes place 72 h after the induction of middle cerebral artery occlusion in the rat. Fenofibrate (50 mg/kg/day) or vehicle was administered by twice-daily gavage over 72 h. Values are means ± SEM. (a) Blinded, histological evaluation of the number of petechiae (as seen in the picture) in the infarct area revealed a significant decrease (^*∗*^ *p* < 0.05) in the hemorrhagic risk of tPA when fenofibrate had been administered (*n* = 7–9 per group). (b) The total, cortical, and striatal infarction volumes were clearly lower in fenofibrate-treated rats, compared with the vehicle group (*n* = 7–9 per group, ^*∗*^ *p* < 0.05) as seen in the histological images for cresyl fast violet staining. (c) The application of increasing doses of acetylcholine led to endothelium-dependent relaxation, which was altered by ischemia and thrombolysis (^*∗*^ *p* < 0.05). However, the postischemia dysfunction in relaxation was prevented by acute treatment with fenofibrate (^#^ *p* < 0.05) (*n* = 5-6 per group). (d) Expression of ICAM-1 adhesion protein, a marker of leukocyte/endothelium interactions, was increased during ischemia and thrombolysis but was lowered by fenofibrate treatment (*n* = 5 per group; ^*∗*^ *p* < 0.05); scale bar, 100 *μ*m. (e) Neutrophil infiltration into the infarct area, quantified by counting the anti-myeloperoxidase-positive cells, was significantly decreased by fenofibrate treatment (*n* = 5 per group; ^*∗*^ *p* < 0.05). Scale bar, 100 *μ*m. (f) Expression of CD11b by Ox 42 antibody, a marker of microglial activation, was increased during ischemia and thrombolysis but was lowered by fenofibrate treatment (*n* = 5 per group; ^*∗*^ *p* < 0.05); scale bar, 25 *μ*m.

In our model, treatment with tPA after MCAO led to intracerebral hemorrhages observed in the infarct area (Figure 1(a)). Fenofibrate administered (50 mg/kg/day) for three days after stroke onset has significantly reduced the number of petechiae (1.78 ± 0.76) compared to vehicle (5.50 ± 0.72, *p* < 0.05).

### 3.2. Effect of Fenofibrate on Infarct Volume

Treatment with fenofibrate resulted in a significant reduction in the total infarct (200.65 ± 29.75 mm^3^ versus 133.72 ± 16.36 mm^3^, *p* < 0.05), in both cortical (148.32 ± 24.07 mm^3^ versus 101.67 ± 16.29 mm^3^, *p* < 0.05) and striatal (52.33 ± 6.77 mm^3^ versus 32.05 ± 1.89 mm^3^, *p* < 0.05) regions (Figure 1(b)).

### 3.3. Effect of Fenofibrate on Cerebrovascular Endothelial Function

Application of increasing Ach doses led to endothelium-dependent relaxation, which was altered by MCAO and administration of tPA (maximal relaxation: 8.77 ± 2% for vehicle group versus 19.98 ± 1% for control group; *p* < 0.05) (Figure 1(c)). This endothelial relaxation dysfunction was significantly prevented by acute administration of fenofibrate for three days after the start of the ischemic episode (maximal relaxation: 42.56 ± 7%; *p* < 0.001).

### 3.4. Effect of Fenofibrate on Neutrophil Infiltration and Microglial Activation

Neutrophil infiltration and microglial activation were induced by MCAO and tPA treatment. Fenofibrate administration decreases the leukocyte/endothelium interactions by lowering the expression of PMN endothelial adhesion proteins ICAM-1 (39 ± 4.2 positive cells in vehicle group versus 3.2 ± 0.7 positive cells in fenofibrate group; *p* < 0.05) (Figure 1(d)) and the parenchymatous infiltration of PMN (85.3 ± 3.3 positive cells in vehicle group versus 14.7 ± 1.6 positive cells in fenofibrate group; *p* < 0.05) (Figure 1(e)). It also prevents the postischemia* in situ* microglial activation (83.3 ± 1.7 positive cells in vehicle group versus 12.2 ± 2.9 positive cells in fenofibrate group; *p* < 0.05) (Figure 1(f)).

## 4. Discussion

We demonstrate for the first time that using a direct PPAR-alpha agonist such as fenofibrate at the acute phase of experimental stroke reduces the occurrence of thrombolysis-associated hemorrhage. This effect is associated with a protective effect on the infarct area, on the endothelial function of the middle cerebral artery, and with a decrease in the inflammatory response induced by ischemia, with reduced microglial activation and neutrophil infiltration in the infarcted area.

Intracerebral hemorrhages after thrombolysis with tPA affect 5 to 6% of treated stroke patients and are associated with high mortality and poor outcome. A better understanding of their pathophysiology is currently necessary for the development of adjuvant strategies aimed at reducing this risk. A large infarct volume and the loss of integrity of cerebral vessels are two of the major contributors of ICH in an ischemic context [7]. It is now well accepted that early reperfusion after ischemia leads to a reduced infarct volume and a reduced risk of postthrombolysis ICH. Although recently suggested [8], the link between stroke and the cerebral vessels integrity is still not fully established but the well-known blood-brain-barrier (BBB) early impairment after ischemia takes part directly in the occurrence of tPA-induced hemorrhage [9]. Many mechanisms have been described at the neurovascular unit to explain the microvascular breakdown process, some of them involving matrix metalloproteinases (MMPs). These enzymes alter the vascular permeability, allowing blood extravasation and the infiltration of peripheral neutrophils into the infarcted area, exposed therefore to exacerbated and deleterious inflammatory response [10]. Moreover, ischemia-induced deprivation of oxygen and glucose to neurons leads to microglial activation, a source among others of MMPs release. In addition, tPA itself promotes leukocyte infiltration and microglial activation, these two phenomena being part of the hemorrhagic risk [11].

Fenofibrate in our model is acting on both parenchymal and vascular tissue, reducing consequently the number of hemorrhages after thrombolysis. Expression of PPAR in neuronal, glial, and vascular cells is consistent with this beneficial effect [12]. Although substantial work is needed to completely describe this protective mechanism, these results strengthen the idea that a protection of both sides of the neurovascular unit from the tPA toxicity in an ischemic context would be necessary for reducing ICH. In this perspective, targeting PPAR-alpha may be a valuable strategy.

In summary, we experimentally demonstrated the benefit of acute administration of fenofibrate in the aftermath of brain ischemia as adjunctive therapy to thrombolysis, to reduce the hemorrhagic risk of tPA. Further studies are warranted to confirm whether PPAR-alpha agonist may be useful to improve stroke management.

## Abbreviations

ICH: Intracerebral hemorrhages
MCAO: Middle cerebral artery occlusion
PMN: Polymorphonuclear neutrophils
PPAR: Peroxisome proliferator-activated receptor
tPA: Tissue plasminogen activator.
